# Investigation of the *STOX1* polymorphism on lumbar disc herniation

**DOI:** 10.1002/mgg3.1038

**Published:** 2019-11-13

**Authors:** Xuejun Yang, Feng Li, Daqi Xin, Zhi Huang, Jianmin Xue, Bo Wang, Yifeng Da, Wenhua Xing, Yong Zhu

**Affiliations:** ^1^ The Second Affiliated Hospital of Inner Mongolia Medical University Hohhot China; ^2^ Inner Mongolia Medical University Hohhot China

**Keywords:** gene expression, lumbar disc herniation, polymorphism, *STOX1*

## Abstract

**Background:**

Lumbar disc herniation (LDH) is a common musculoskeletal disorder affliction and associated with several genes polymorphism. Storkhead box 1 (*STOX1*) gene is a transcriptional factor related with several signaling pathways including inflammatory pathway. However, little is known about single‐nucleotide polymorphisms (SNPs) of STOX1 associated with LDH risk.

**Methods:**

We conducted a case–control study among 508 LDH cases and well‐matched 508 controls, and six candidate SNPs in *STOX1* were genotyped by Agena MassARRAY. Chi‐squared test, genetic model, and haploview analysis were used to evaluate associations. Odds ratios (ORs) and 95% confidence intervals (CIs) were calculated by unconditional logistic regression.

**Results:**

In the allelic model analysis, we found the minor allele “T” of rs7903209 and “A” of rs4472827 were associated with an increased risk of LDH (*p* = .029, *p* = .016). Furthermore, in the genotype model analysis, rs7903209 polymorphism was associated with the increased susceptibility of LDH based on dominant (*p* = .033) and additive model (*p* = .024); and rs4472827 variant was found to play a harmful role in the LDH risk based on genotype (*p* = .014), dominant (*p* = .012), and additive model (*p* = .015). In the haplotype analysis, the haplotype “GT” in block (rs10998461 and rs10998468) decreased LDH risk (OR = 0.7, 95% CI = 0.52–0.93, *p* = .016). Functional assessment indicated that rs7903209 and rs4472827 polymorphisms may influence the expression of *STOX1*.

**Conclusion:**

Our results provide evidence for polymorphisms of rs7903209 and rs4472827 in *STOX1* associated with LDH risk in Chinese Han population.

## Graphical abstract

We aimed to explore the association between STOX1 polymorphisms and LDH risk, and we found that STOX1 might be a functional factor for the occurrence and development of LDH.

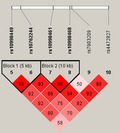

## 1 BACKGROUND

Low back and leg pain is the main characteristic of lumbar disc herniation (LDH), which is the result of lumbar disc degeneration and nucleus pulposus protrusion from the defective anulus fibrosus compressing the spinal nerve root (Han et al., 2015). The sciatica is the most prevalent symptom of symptomatic LDH, and approximately 90% of the time, symptomatic LDH induces the sciatica. Many people will experience low back and leg pain at some point in their lives, and LDH explains about 50% of low back and leg pain problems (Karamouzian et al., 2014). As a common, complex, and multifactorial spine disease, LDH is influenced by diverse factors, including humid or cold environment, external injury, and genetic factors. Although the determinants of the pathogenesis on LDH are unclear, genetic factors are increasingly recognized as a meaningful role in the pathogenesis of LDH (Battie, Videman, Levalahti, Gill, & Kaprio, 2007; Rajasekaran et al., 2015; Zhang, Sun, Liu, & Guo, 2008).

Most recently, molecular epidemiological researches have marked the potential and important role of polymorphisms in genes correlated with LDH. According to Gyda Bjornsdottir1 et al. (Bjornsdottir et al., 2017), 37 highly correlated sequence variants located at 8q24.21 have been found associated with LDH‐induced sciatica based on genome‐wide association study (GWAS). Liu S et al. reported that single‐nucleotide polymorphisms (SNPs) rs4233367 in the exon of *ADAMTS4* gene associated with a decreased LDH risk in Chinese Han population (Liu et al., 2016). Jiang H et al. indicated that rs1337185 in *COL11A1* and rs162509 in *ADAMTS5* have been associated with LDH, and rs1337185 has been found as a risk factor for LDH pathologies (Jiang, Yang, Jiang, Zhan, & Xiao, 2017). In addition, polymorphism in *GPR126* was demonstrated as a functional correlation with LDH risk in Chinese population (Qin et al. 2017). These studies have offered a strong association between LDH and genetic factors, but the researches about gene polymorphisms in LDH risk at present are far from enough.

Storkhead box 1 (*STOX1*) gene (OMIM: 609,397) is located in 10q22.1, and STOX1 protein functions as a DNA‐binding protein. Previously, *STOX1* was structurally and functionally related to the forkhead fox protein, which plays a role in the regulation of genes involved in multiple disease associated pathway (Benayoun, Caburet, & Veitia, 2011; Tuteja & Kaestner, 2007). Furthermore, this study has identified that *STOX1* could play an oncosuppressing effect via direct transcription repression (Zhang et al., 2016). However, no studies have explored the correlation between *STOX1* polymorphisms and LDH risk. Our case–control study investigated whether *STOX1* has a potential association with LDH risk among a population of Chinese at a genetic level.

## 2 MATERIAL AND METHODS

### 2.1 Study participants

A total of 508 patients with LDH and 508 controls were enrolled in the study. All the patients were recruited from the Second Affiliated Hospital of Inner Mongolia Medical University and the Hohhot First Hospital. The eligible patients were confirmed with degenerative discs of the lumbar spine by computed radiography, computed tomography, and/or magnetic resonance imaging scan between 2016 and 2018. According to the Pfirrmann grading system, grades 1 and 2 indicate a normal disc, and grade 3 or above are evaluated as LDH (Pfirrmann, Metzdorf, Zanetti, Hodler, & Boos, 2001). Patients with complicated blood diseases, tumors, trauma, rheumatoid arthritis, and related lumbar spine disease were excluded from this study. The controls were healthy people from the medical examination during the same period and had no history of sciatica and low back pain. Individuals exposed to known environmental risk factors, including heavy smoking and heavy manual labor, were excluded. Written informed consents were obtained from each participant before this study. The Ethics Committee of the Second Affiliated Hospital of Inner Mongolia Medical University and the Hohhot First Hospital approved this study.

### 2.2 SNP selection and Genotyping

In this study, six SNPs (rs10998449, rs10762244, rs10998461, rs10998468, rs7903209, and rs4472827) in the *STOX1* were selected from the DbSNP ( http://www.ncbi.nlm.nih.gov/projects/SNP/ ) and 1,000 genome ( http://www.internationalgenome.org/ ). All the SNPs were selected at a minor allele frequency >5% in Han Chinese from the 1,000 Genome Projects.

According to the manufacturer's protocol, GoldMag‐Mini Whole Blood Genomic DNA Purification Kit (GoldMag Co. Ltd.) was used to isolate the total genomic DNA from peripheral blood. The Agena Bioscience Assay Design Suite V2.0 software (http://agenacx.com/online-tools) was used to design the extended primer. The MassARRAY Nanodispenser (Agena Bioscience) and MassARRAY iPLEX platform (Agena Bioscience) were used to genotype, and the Agena Bioscience TYPER software (version 4.0) was used to analyze the data. We randomly selected about 10% of the sample to repeat genotyping, and the reproducibility was 100% indicating that our result is reliable.

### 2.3 Data analysis

SPSS 19.0 (SPSS, lnc., Chicago, IL, USA) was used to perform data analysis. All *p*‐ values were two‐sided, and *p* ≤ .05 indicated a significant difference. The Hardy–Weinberg equilibrium (HWE) was checked for all polymorphisms in both the control and the patient groups using Fisher's exact tests. The difference in allelic and genotype frequency distribution of each SNP between patients with LDH and controls was assessed by Pearson's Chi‐squared test. Odds ratios (OR) and 95% confidence intervals (CI) were calculated to estimate the association between *STOX1* and the LDH risk using unconditional logistic regression analysis with or without adjustment for potential confounding. In this study, the wild‐type allele was regarded as a reference. Four genotype models (genotype, dominant, recessive, and additive model) were applied by PLINK software (http://www.cog-genomics.org/plink2/) to assess the association between SNP and LDH risk. Finally, Haploview software package (version 4.2) and the SHEsis software (http://analysis.bio-x.cn/myAnalysis.php) were used to construct the pairwise linkage disequilibrium (LD), haplotype, and genetic association of polymorphism loci (Barrett, Fry, Maller, & Daly, 2005; Shi & He, 2005).

## 3 RESULTS

### 3.1 Characteristics of patients and controls

In this study, we collected and analyzed 508 cases of LDH and 508 healthy controls (Table 1). The two groups were exact match on gender distribution (*p* = 1), and male were 297 and female were 211 in each group. The mean ages of the patients and the controls were 48.49 ± 13.71 and 49.16 ± 14.91, and the *p*‐value was .457. In males, the mean age of the patients and the controls were 46.89 ± 14.07 and 47.71 ± 15.09, the *p*‐value was .493. In females, the mean age of the patients and the controls were 50.74 ± 12.89 and 51.20 ± 14.43, and the *p*‐value was .733. The above statistical results indicated that the cases and the control were well‐matched.

**Table 1:** Characteristic variables in LDH cases and control patients

Characteristic	Case	Control	*p*‐value
Gender (%)			1^a^
Male	297 (58.46%)	297 (59.46%)	
Female	211 (41.54%)	211 (41.54%)	
Age (Mean age ± *SD*, years)			
Whole	48.49 ± 13.71	49.16 ± 14.91	.457^b^
Male	46.89 ± 14.07	47.71 ± 15.09	.493^b^
Female	50.74 ± 12.89	51.20 ± 14.43	.733^b^

Abbreviation: LDH, lumbar disc herniation.

a *p*‐value was calculated using two‐sided Chi‐squared test.

b *p*‐value was calculated using independent samples *t* test.

### 3.2 Allelic analysis

We genotyped six SNPs in the *STOX1*, and all the SNPs were in HWE (Table 2). Among the six SNPs, only rs7903209 and rs4472827 showed significant allelic difference between case and control group (*p* = .029, *p* = .016, respectively). The minor allele “T” of rs7903209 was prevalent in case group and played a harmful role in LDH patients (OR = 1.36, 95% CI = 1.04–1.79). Similarly, the minor allele “A” of rs4472827 was associated with an increased risk of LDH (OR = 1.46, 95% CI = 1.08–1.96).

**Table 2:** Candidate SNPs examined in the *STOX1*

SNP_ID	Chromosome	Position	Allele (A/B)	Minor allele frequency		*p*‐HWE^a^	OR	95% CI	*p* b
				Case	Control				
rs10998449	13	93239636	T/C	0.264	0.277	.506	0.94	0.77–1.14	.549
rs10762244	13	93243089	G/A	0.250	0.257	.103	0.96	0.79–1.18	.760
rs10998461	13	93250331	T/G	0.382	0.406	.461	0.90	0.76–1.08	.276
rs10998468	13	93263477	T/C	0.496	0.488	.656	1.03	0.87–1.23	.722
rs7903209	13	93263913	T/C	0.136	0.103	.809	1.36	1.04–1.79	.029*
rs4472827	13	93356953	A/G	0.112	0.079	.355	1.46	1.08–1.96	.016*

Abbreviation: A, minor allele; B, major allele; CI, confidence interval; HWE, Hardy–Weinberg equilibrium; OR, odds ratio; SNP, single‐nucleotide polymorphism.

a *p*‐value was calculated using exact test.

b *p*‐value was calculated using two‐sided Chi‐squared test.

*p < .05 indicated a statistical significance.

### 3.3 Genotypic analysis

The genotypic analysis showed significant association between SNPs rs7903209 and rs4472827 and LDH risk (Table 3). We found that rs7903209 was related to an increased risk of LDH based on dominant model (“C/T‐T/T” vs. “C/C” OR = 1.38, 95% CI = 1.02–1.85, *p* = .035) and additive model (OR = 1.36, 95% CI = 1.04–1.77, *p* = .026) without adjustment. And, after adjustment for compounding factor, the significant association was still based on dominant model (“C/T‐T/T” vs. “C/C” OR = 1.38, 95% CI = 1.03–1.86, *p* = .033) and additive model (OR = 1.36, 95% CI = 1.04–1.78, *p* = .024). Rs4472827 was observed to be associated with enhanced susceptibility of LDH risk in genotype model (“G/A” vs. “G/G” OR = 1.51, 95% CI = 1.09–2.11, *p* = .015), dominant model (“G/A‐A/A” vs. “G/G” OR = 1.51, 95% CI = 1.1–2.09, *p* = .012), and additive model (OR = 1.44, 95% CI = 1.07–1.94, *p* = .016) without adjustment. Furthermore, after adjustment for compounding factor, rs4472827 variant was also related with the LDH risk in genotype model (“G/A” vs. “G/G” OR = 1.52, 95% CI = 1.09–2.12, *p* = .014), dominant model (“G/A‐A/A” vs. “G/G” OR = 1.52, 95% CI = 1.1–2.1, *p* = .012), and additive model (OR = 1.45, 95% CI = 1.07–1.94, *p* = .015).

**Table 3:** Association of prominent SNPs with the LDH risk under genotypic model

SNP	Model	Genotype	Group = case	Group = control	Without adjustment		Adjustment analysis	
					OR (95% CI)	*p* a	OR (95% CI)	*p* b
rs7903209	Genotype	C/C	381 (75%)	409 (80.51%)	1		1	
		C/T	116 (22.83%)	93 (18.31%)	1.34 (0.99–1.82)	.062	1.34 (0.99–1.83)	.059
		T/T	11 (2.17%)	6 (1.18%)	1.97 (0.72–5.37)	.187	1.99 (0.73–5.42)	.181
	Dominant	C/C	381 (75%)	409 (80.51%)	1		1	
		C/T‐T/T	127 (25%)	99 (19.49%)	1.38 (1.02–1.85)	.035*	1.38 (1.03–1.86)	.033*
	Recessive	C/C‐C/T	497 (97.83%)	502 (98.82%)	1		1	
		T/T	11 (2.17%)	6 (1.18%)	1.85 (0.68–5.05)	.228	1.87 (0.68–5.09)	.223
	Additive	—	—	—	1.36 (1.04–1.77)	.026*	1.36 (1.04–1.78)	.024*
rs4472827	Genotype	G/G	401 (78.94%)	431 (85.01%)	1		1	
		G/A	100 (19.69%)	71 (14%)	1.51 (1.09–2.11)	.015*	1.52 (1.09–2.12)	.014*
		A/A	7 (1.38%)	5 (0.99%)	1.51 (0.47–4.78)	.488	1.52 (0.48–4.82)	.48
	Dominant	G/G	401 (78.94%)	431 (85.01%)	1		1	
		G/A‐A/A	107 (21.06%)	76 (14.99%)	1.51 (1.1–2.09)	.012*	1.52 (1.1–2.1)	.012*
	Recessive	G/G‐G/A	501 (98.62%)	502 (98.82%)	1		1	
		A/A	7 (1.38%)	5 (0.98%)	1.40 (0.44–4.45)	.566	1.41 (0.45–4.49)	.557
	Additive	—	—	—	1.44 (1.07–1.94)	.016*	1.45 (1.07–1.94)	.015*

Abbreviation: CI, Confidence interval; LDH, lumbar disc herniation; OR, Odds ratio; SNP, single‐nucleotide polymorphism.

a *p*‐values were calculated by unconditional logistic regression analysis without adjustment.

b *p*‐values were calculated by unconditional logistic regression analysis with adjustment for confounding factor.

*p < .05 indicates statistical significance.

### 3.4 Haplotype analysis

**Figure 1:**
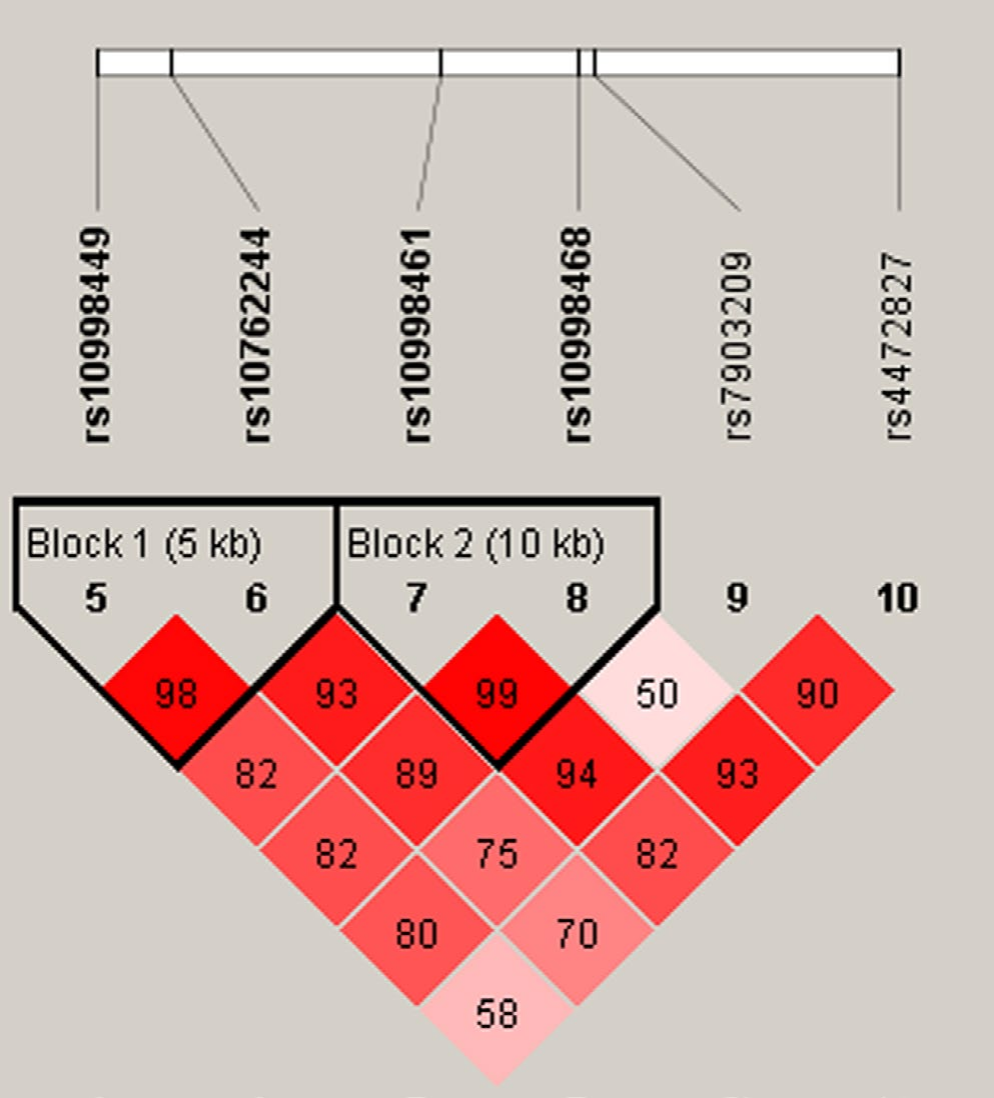
Haplotype block map for part of the SNPs in the *STOX1*. LD plots containing six SNPs in *STOX1*, and standard color frame is used to show LD pattern. Two blocks in the figure showed higher LD, and D’ values were 0.98 and 0.99, respectively

Our study used polymorphism detection to analyze the pairwise LD of STOX1. The parameters r2 and D’ were used to analyze the LD pattern, and the results are shown in Figure 1 and Table 4. We observed two blocks in STOX1, including rs10998449|rs10762244 and rs10998461|rs10998468. We used Chi‐squared and logistic tests adjusted by compounding factor to analyze the haplotype (Table 4). The haplotype “GT” was found to be prevalent in the control group (*p* = .014), and was associated with a significantly decreased LDH risk (OR = 0.7, 95% CI = 0.52–0.93, *p* = .016). As for other haplotypes, we did not find any association between them and LDH risk.

**Table 4:** *STOX1* haplotype frequencies and the association with LDH risk

Block	Haplotype	Freq (case)	Freq (control)	*p* a	OR (95% CI)	*p* b
rs10998449|rs10762244	CG	0.250	0.256	.760	0.97 (0.79–1.18)	.745
	TA	0.736	0.724	.549	1.06 (0.87–1.29)	.542
	CA	0.514	0.533	.399	0.93 (0.78–1.1)	.386
rs10998461|rs10998468	TT	0.62	0.595	.253	1.11 (0.93–1.33)	.248
	GT	0.884	0.917	.014*	0.7 (0.52–0.93)	.016*
	GC	0.502	0.511	.687	0.97 (0.81–1.15)	.694

a *p*‐values were calculated by two‐sided Chi‐squared test.

b *p*‐values were calculated by unconditional logistic regression analysis with adjustment for confounding factor.

*p < .05 indicates statistical significance.

### 3.5 Functional assessment

The biological effects of rs7903209 and rs4472827 variants on *STOX1* expression were assessed using the genotype‐tissue expression (GTEx) database of quantitative trait loci (eQTL) variant (http://www.gtexportal.org). The results are listed in Table 5. We found that rs7903209 variant significantly affected the expression of *STOX1* in muscle‐skeletal, adrenal gland, esophagus‐mucosa, pancreas, whole blood, esophagus‐muscularis, testis, and nerve‐tibial. Rs4472827 variants significantly affected the expression of *STOX1* in muscle‐skeletal, adrenal gland, esophagus‐mucosa, lung, esophagus‐muscularis, and colon‐transverse. These results served a powerful approach to uncover the two SNPs underlying altered gene expression.

**Table 5:** Association between LDH‐related SNPs and *STOX1* expression

SNP	NES	*p*‐value	Tissue
rs7903209	0.46	5.6 × 10^−19^	Muscle‐Skeletal
rs7903209	0.88	3.9 × 10^−19^	Adrenal Gland
rs7903209	0.4	1.4 × 10^−11^	Esophagus‐Mucosa
rs7903209	0.48	8.5 × 10^−10^	Colon‐Transverse
rs7903209	0.58	6.9 × 10^−9^	Pancreas
rs7903209	0.43	1.7 × 10^−7^	Whole Blood
rs7903209	0.24	2.6 × 10^−7^	Esophagus‐Muscularis
rs7903209	0.42	4.1 × 10^−7^	Testis
rs7903209	0.28	1.9 × 10^−6^	Nerve‐Tibial
rs4472827	0.35	1.2 × 10^−12^	Muscle‐Skeletal
rs4472827	0.57	7.8 × 10^−9^	Adrenal Gland
rs4472827	0.32	1.6 × 10^−8^	Esophagus‐Mucosa
rs4472827	0.18	1.1 × 10^−6^	Lung
rs4472827	0.21	1.6 × 10^−6^	Esophagus‐Muscularis
rs4472827	0.33	2.0 × 10^−5^	Colon‐Transverse

Abbreviation: LDH, lumbar disc herniation; NES, normalized effect size; SNP, single‐nucleotide polymorphism.

## 4 DISCUSSION

In this case–control study, two novel SNPs rs7903209 and rs4472827 in the *STOX1* were statistically significantly associated with an increased risk of LDH. We also observed that a haplotype “GT” of *STOX1* was associated with a 30% reduction in the risk of LDH. In silico analysis of SNPs revealed that rs7903209 and rs4472827 may play an important role in the occurrence and development of LDH via regulating the expression of the *STOX1*. These findings suggest that polymorphisms of *STOX1* may influence the risk of LDH among North Chinese individuals.

*STOX1*, a gene mapped in chromosome 10q22.1, is also known as *C10orf24*, which is expressed in several tissues, including adrenal, brain, and testis so on. *STOX1* has two isoforms expression STOX1A and STOX1B, while only STOX1A is supposed to play a role in gene activation via uniting with the transcription factor‐binding site. Research have indicated that *STOX1* has multiple gene targets, especially in pathways connected to oxidative stress, cell cycle, and inflammation (Vaiman & Miralles, 2016). Doridot L et al. have found that STOX1 can play a genetic switch in the nitroso–redox balance and mitochondrial homeostasis (Doridot et al., 2014). Furthermore, STOX1 was also found to involve inner ear epithelial cell proliferation as a novel stimulatory factor activated by phosphorylation of protein kinase B pathway (Nie et al., 2015). LDH is a degenerative disease related to narrowing of the spinal canal or intervertebral foramina. Studies have found that various inflammatory‐related factors play a crucial role in lumbar disc degeneration and nervous radical pain (Hoyland, Le Maitre, & Freemont, 2008; Liu, Jin, Shen, Balian, & Li, 2013). Moreover, some publications also proved that patients with LDH have a higher pro‐inflammatory factor expression level than the healthy, and these pro‐inflammatory factors conversely quicken degenerative severity via elevating extracellular matrix breakdown (Phillips et al., 2015; Weber et al., 2016). Therefore, we propose a reasonable hypothesis that the pathogenesis of LDH is correlated with *STOX1*, and *STOX1* may involve in the occurrence and development of LDH via changing the inflammatory response.

Many earlier studies have identified that *STOX1* was found to be the first gene associated with preeclampsia susceptibility (George & Bidwell, 2013; Kivinen et al., 2007). And, this study has reported that overexpression of STOX1 in placenta induced a switch between nitrosative and oxidative stress (Doridot et al., 2013) and intrauterine growth restriction (Collinot et al., 2018). Furthermore, *STOX1* was found to be overexpressed and associated with Alzheimer's disease, and studies have shown that *STOX1* may control a conserved pathway shared between the placenta and the brain (van Dijk et al., 2010). In addition, *STOX1* was identified as a transcriptional suppressor that has a pivotal role in the cerebellar granule neurogenesis and medulloblastoma formation (Zhang et al., 2016). However, we have not found any evidence for the role of heredity between *STOX1* and LDH susceptibility in previous studies. In the present study, six *STOX1* SNPs were genotyped, and rs7903209 and rs4472827 were singled out to be relatively associated with LDH risk. Carriers of the rs7903209 “T” allele exhibited a statistically significant increased 1.36‐, 1.38‐, and 1.36‐fold LDH susceptibility by the allelic, dominant, and additive model, respectively. As for rs4472827, the risk was 1.46‐, 1.52‐, 1.52‐, and 1.45‐fold LDH susceptibility by allelic, genotypic, dominant, and additive model, respectively. Haplotype‐based association recovered that haplotype “GT” of rs10998461|rs10998468 block exhibited a protective role in the LDH susceptibility.

As for the present study, some limitations that may bias our findings should be taken into consideration. First, all participants were recruited from the same hospital. Secondly, the number of cases in our study was not large, and the study participants were limited in Inner Mongolia. Therefore, the inherent selection bias cannot be excluded and extended to other nations. Therefore, a larger sample size and further confirmation in other ethnic groups are needed for further validation. We will continue to investigate the association between *STOX1* polymorphisms and LDH risk in other geographical area.

## 5 CONCLUSION

In conclusion, the results of our case–control study provide evidence for the polymorphisms rs7903209 and rs4472827 in the *STOX1* associated with LDH risk in Chinese Han population, and *STOX1* has also been identified as a common LDH susceptibility gene for the first time. Our study may provide new data for screening of LDH in Han Chinese population and shed light on the new candidate genes and new ideas for the mechanism of LDH. To assess its role further, genetic tests in other population groups may be needed with subsequent research to increase power for our findings.

## CONFLICT OF INTEREST

The authors declare that there are no conflict of interest.

## AUTHOR CONTRIBUTIONS

XJY and FL: Write and revise manuscripts; DQX: performed the experiments; ZH and JMX: analyzed the data; BW and DYF: contributed reagents/materials/analysis tools; WHX and YZ: conceived and designed the experiments. All authors contributed significantly to the final draft of the paper and agreed to submit the manuscript for publication.
